# Association between delayed ambulation and increased risk of adverse events after lumbar fusion surgery in elderly patients

**DOI:** 10.1186/s12891-024-07606-8

**Published:** 2024-06-27

**Authors:** Shuai-Kang Wang, Xin-Yi Chai, Peng Wang, Chao Kong, Shi-Bao Lu

**Affiliations:** 1 Department of Orthopedics & Elderly Spinal Surgery, National Clinical Research Center for Geriatric Diseases, Xuanwu Hospital of Capital Medical University, No.45 Changchun Street, Xicheng District, Beijing, 100053 China; 2 National Clinical Research Center for Geriatric Diseases, Beijing, 10053 China; 3 Capital Medical University, Beijing, 10053 China

**Keywords:** Lumbar degenerative disease, Transforaminal lumbar interbody fusion, Delayed ambulation, Enhanced recovery after surgery, Adverse events

## Abstract

**Purpose:**

The relationship between delayed ambulation (DA) and postoperative adverse events (AEs) following transforaminal lumbar interbody fusion (TLIF) in elderly patients remains elusive. The aim of our study was to evaluate the effects of DA on the postoperative AEs including complications, readmission and prolonged length of hospital stay (LOS).

**Methods:**

This was a retrospective analysis of a prospectively established database of elderly patients (aged 65 years and older) who underwent TLIF surgery. The early ambulation (EA) group was defined as patients ambulated within 48 h after surgery, whereas the delayed ambulation (DA) group was patients ambulated at a minimum of 48 h postoperatively. The DA patients were 1:1 propensity-score matched to the EA patients based on age, gender and the number of fused segments. Univariate analysis was used to compare postoperative outcomes between the two groups, and multivariate logistic regression analysis was used to identify risk factors for adverse events and DA.

**Results:**

After excluding 125 patients for various reasons, 1025 patients (≤ 48 h: *N* = 659 and > 48 h: *N* = 366) were included in the final analysis. After propensity score matching, there were 326 matched patients in each group. There were no significant differences in the baseline data and the surgery-related variables between the two groups (*p* > 0.05). The patients in the DA group had a significant higher incidence of postoperative AEs (46.0% vs. 34.0%, *p* = 0.002) and longer LOS (*p* = 0.001). Multivariate logistic regression identified that age, operative time, diabetes, and DA were independently associated with postoperative AEs, whereas greater age, higher international normalized ratio, and intraoperative estimated blood loss were identified as independent risk factors for DA.

**Conclusions:**

Delayed ambulation was an independent risk factor for postoperative AEs after TLIF in elderly patients. Older age, increased intraoperative blood loss and worse coagulation function were associated with delayed ambulation.

## Introduction

Low back pain was remaining in the top-ten-ranking causes of disability adjusted life years from 1990 to 2019 [1]. Lumbar degenerative disease is a common age-related musculoskeletal disorder which is a major cause of chronic low back pain and the pain-related disabilities [2]. For patients who have failed to respond to non-operative treatment, transforaminal lumbar interbody fusion (TLIF) is an effective treatment to improve the stability of the spine and relieve low back pain and concomitant radicular pain[3, 4]. From 2004 to 2015, the volume of elective lumbar fusion procedures among those over age 65 increased by 138% and the costs for elective lumbar fusion increased by 177.2% from $3.7 billion dollars in 2004 to $10.2 billion dollars in 2015 [4]. Adverse events (AEs) following fusion surgery include complications, prolonged length of hospital stay (LOS) and readmission, which increase hospitalization-related expenditures and postoperative dissatisfaction. In traditional postoperative care pathways, immobilization or bed rest after fusion surgery is often recommended, which can hinder the postoperative rehabilitation process [6, 7]

Enhanced recovery after surgery (ERAS) is an evidence-based, multidisciplinary, perioperative management pathway which aims to reduce surgery-related stress response and accelerates postoperative rehabilitation[8]. Early ambulation (EA), avoiding prolonged fasting and early removal of tube are three key elements of the ERAS program [9, 10]. Prolonged bed rest and reduced mobility after TLIF increase risk of postoperative complications including deep vein thrombosis, gastrointestinal dysfunction, and infection [11]. Although the importance of early rehabilitation has been described in many studies, low adherence to early ambulation remains a shortcoming in the implementation of ERAS programs. Pain and restraining medical devices, such as catheters and intravenous lines, and patient and provider concerns about complications are common causes of bed rest after surgery.

The elderly patients are less likely to recover from TLIF surgery rapidly compared with younger patients[11, 12] However, there is still a lack of research on the relation between delayed (DA) and postoperative AEs in elderly patients. The aim of our study was to evaluate the effects of DA on elderly patients underwent TLIF.

## Method

### Patient selection

This was a single-center retrospective analysis of a prospectively established database of elderly patients (≥ 65 years old) who underwent TLIF surgery. Consecutive patients who underwent TLIF surgery for lumbar degenerative disease between January 2019 and January 2023 were included. The exclusion criteria were as follows: patients with (i) irreversible loss of mobility before admission; (ii) severe cognitive impairment; (iii) incomplete data; (iv) preexisting spinal fracture, any spinal infection or any malignancy; and (v) early ambulation was contraindicated due to complications (durotomy or suspected cerebrospinal fluid leak). This study was approved by the ethical review committee of Xuanwu Hospital, Capital Medical University (IRB# 2,018,086). Due to the nature of this retrospective study, the informed consent from patients was waived remitted.

### Ambulation status

Since the implementation of the enhanced recovery after lumbar surgery protocol at our center in January 2019, all patients have been educated about the importance of early ambulation and avoiding extended bed rest [13]. To date, there are no mobilization guidelines for adults undergoing elective spinal surgery [14]. To increase compliance with the early ambulation protocol of ERAS pathway, a care bundle was implemented in spinal fusion surgery in our center from 2019. Based on previous evidences and consultation, the care bundle based in five components was implemented: (1)preoperative education on the importance of early ambulation and avoiding extended bed rest; (2) goal-directed fluid management; (3) early removal of all catheter; (4) nutritional support and exercises for the lower extremity muscles; (5) multimodal analgesia[15–17]. Ambulation time was recorded when the patient was up and walking any distance (either assisted or unassisted), including walking to the chair from bed. Patients who ambulated within 48 h after surgery were grouped into EA group, whereas patients who ambulated after more than 48 h after surgery were grouped into DA group.

### Data collection

We extracted all the data from the medical record as well as the electronic medical record system for inpatients. Preoperative baseline data included demographic variables (age, gender, weight, and body mass index), medical disease (charlson comorbidity index, cardiovascular, diabetes, use of glucocorticoids and anticoagulant agent, osteoporosis, current smoker and drinker), and laboratory test (red blood cell count [RBC], hemoglobin, and international normalized ratio [INR]). Surgery-related variables included number of fused segments, estimated blood loss (EBL), operative time, and drainage volume of postoperative day 0 (POD0). Postoperative AEs included postoperative complications, prolonged LOS, and readmission within 90 days after surgery. Complications were categorized as medical or surgical. Surgical complications included hematoma, surgical site infection, and displacement of implant. Medical complications included urinary retention, deep vein thrombosis, nausea/vomiting, urinary infection, constipation, acute cerebral infarction, pneumonia, delirium, myocardial infarction, heart failure, arrhythmias. Prolonged LOS was defined as an inpatient hospital stay longer than the 75th percentile of LOS.

### Statistical analysis

All statistical analyses were performed by the SPSS software (SPSS, version 22.0, Inc., Chicago, IL, USA). Continuous variables are expressed as the mean and standard deviation, and analyzed by the Student’s *t*-test for normally distributed variables and the Mann–Whitney U test for non-normally distributed variables. Categorical variables are expressed as frequencies with percentages and analyzed using the Fisher’s exact or chi-square tests. A p value of 0.05 was considered significant. The Propensity score matching (PSM) method was used to construct paired matched samples of the EA and DA groups based on age, gender and the number of fused segments to balance confounding variables. The DA patients were 1:1 matched with the EA patients. Variables with a p value < 0.1 in univariate analyses were further included in multivariate analyses. Multivariable logistic regression analysis was used to identify independent risk factors for postoperative AEs and DA.

## Results

### Descriptive analyses

**Fig. 1:**
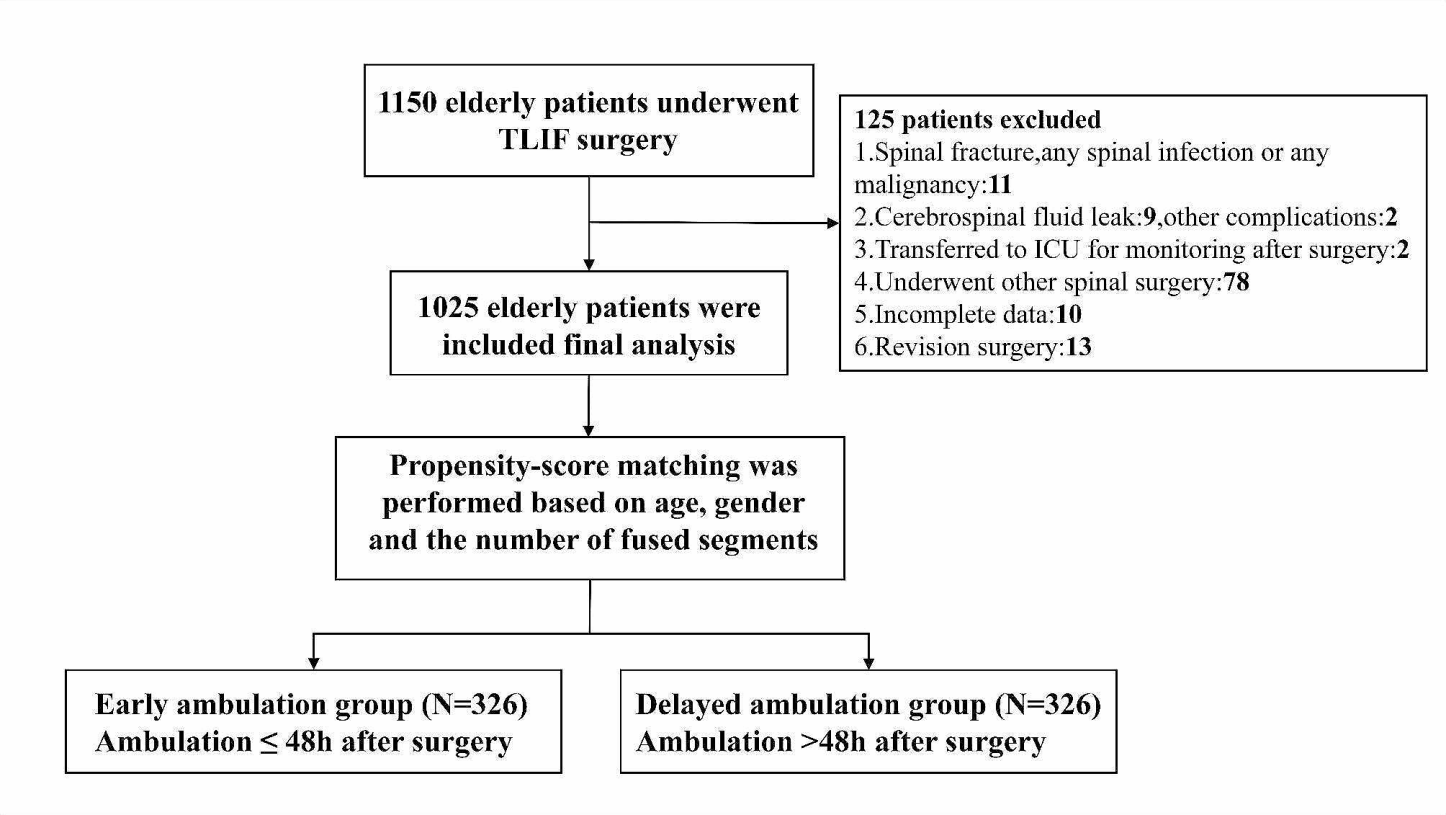
Flow chart of study participants

A total of 1150 elderly patients with TLIF surgery were reviewed in this study. After excluding 125 patients for various reasons, 1025 patients (≤ 48 h: *N* = 659 and > 48 h: *N* = 366) were included in the final analysis (Fig. 1). Age was significantly different between the two groups (EA group: 72.0 ± 5.4, DA group: 74.1 ± 5.8, *p* < 0.05), whereas gender, weight and BMI were similar between the two groups. There were higher rates of coronary heart disease, current smoker, and current drinker in patients of DA group (Table 1). As for surgery-related variables, more fused segments, operative time, intraoperative blood loss, and drainage volume on POD0 were observed in DA group than EA group (Table 2).

**Table 1:** Demographic data, medical diseases, and laboratory test before matching

	EA group	DA group	*P*
Variables	*N* = 659	*N* = 366	Value
*Demographic data*			
Age (yr)	72.0 ± 5.4	74.1 ± 5.8	< 0.05*
male n(%)	267 (40.5%)	126 (34.4%)	0.055
Weight (kg)	67.2 ± 10.9	67.2 ± 11.0	0.664
BMI (kg/m^2^)	25.5 ± 3.6	25.7 ± 3.7	0.435
*Medical disease n(%)*			
CCI			0.520
0 or1	544 (82.5%)	299 (81.7%)	
2 or 3	112 (17.0%)	67 (18.3%)	
4 or more	3 (0.5%)	0	
Hypertension	419 (63.6%)	231 (63.1%)	0.882
Coronary heart disease	127 (19.3%)	93 (25.4%)	0.022*
Diabetes	215 (32.6%)	122 (33.3%)	0.817
Cerebrovascular disease	72 (10.9%)	29 (7.9%)	0.122
Osteoporosis	74 (11.2%)	52 (14.2%)	0.164
Smoker	97 (14.7%)	29 (7.9%)	0.001*
Drinker	65 (9.9%)	20 (5.5%)	0.014*
*Laboratory test*			
Red blood cell count(×10^12^/L)	4.3 ± 0.5	4.2 ± 0.5	0.405
Hemoglobin(g/L)	130.4 ± 15.1	129.7 ± 14.1	0.484
INR	0.97 ± 0.09	0.99 ± 0.1	0.001*
*Medication history*			
Glucocorticoids	11 (1.7%)	5 (1.4%)	0.708
Anticoagulant agent	130 (19.7%)	59 (16.1%)	0.154

DA, delayed ambulation; BMI, Body mass index; CCI, Charlson comorbidity index; INR, International normalized ratio

*Represents for statistically different (*P* < 0.05)

**Table 2:** Surgery-related variables by postoperative ambulation timing before matching

	EA group	DA group	*P*
Variables	*N* = 659	*N* = 366	Value
Operative time (min)	201.3 ± 61.0	229.4 ± 72.7	< 0.001*
Number of fused segments			< 0.001*
1	239 (36.3%)	97 (26.5%)	
2	311 (47.2%)	113 (36.3%)	
3	81 (12.3%)	81 (22.1%)	
4	19 (2.9%)	39 (10.7%)	
5	9 (1.4%)	16 (4.4%)	
Intraoperative EBL (ml)	299.2 ± 286.6	447.4 ± 356.2	< 0.001*
Drainage volume on POD0 (ml)	104.4 ± 78.8	130.5 ± 110.9	< 0.001*

EBL, estimated blood loss; POD0: postoperative day 0

*Represents for statistically different (*P* < 0.05)

After matching, a total of 652 patients (EA group: *n* = 326, DA group: *n* = 326) were matched in this study. There were no significant differences in surgery-related variables and baseline data including demographic variables, medical disease, and laboratory test between the two groups (Table 3). Patients in the DA group have a significant higher incidence of postoperative complications (28.8% vs. 16.0%, *p* < 0.001), especially for medical complications (23.6% vs. 13.8, *p* = 0.002). Compared with the DA group, patients in the EA group also had a significantly shorter hospital length of stay (Table 4).

**Table 3:** Baseline data and surgery-related variables after matching

	EA group	DA group	*P*
Variables	*N* = 326	*N* = 326	Value
*Demographic data*			
Age(yr)	73.1 ± 5.5	73.5 ± 5.6	0.304
male n(%)	116 (35.6%)	117 (35.9%)	1.000
Weight (kg)	66.9 ± 10.6	67.5 ± 11.2	0.479
BMI(kg/m^2^)	25.6 ± 3.5	25.8 ± 3.8	0.635
*Medical disease n(%)*			
CCI			0.681
0 or1	271 (83.1%)	266 (81.6%)	
2 or 3	55 (16.9%)	60 (18.4%)	
Hypertension	215 (66.0%)	203 (62.3%)	0.369
Coronary heart disease	68 (20.9%)	83 (25.5%)	0.194
Diabetes	106 (32.5%)	110 (33.7%)	0.803
Cerebrovascular disease	35 (10.7%)	25 (7.7%)	0.222
Osteoporosis	47 (14.4%)	41 (12.6%)	0.567
Smoker	42 (12.9%)	28 (8.6%)	0.100
Drinker	33 (10.1%)	19 (5.8%)	0.059
*Laboratory test*			
Red blood cell count(×10^12^/L)	4.2 ± 0.5	4.3 ± 0.5	0.266
Hemoglobin (g/L)	128.9 ± 15.1	131.2 ± 15.0	0.079
INR	0.97 ± 0.1	0.98 ± 0.1	0.298
*Surgery-related variables*			
Number of fused segments 1–2 3–5	220(67.5%) 106(32.5%)	230(70.6%) 96(29.4%)	0.466
Operative time (min)	228.9 ± 60.0	222.3 ± 71.1	0.200
Intraoperative EBL (ml)	374.1 ± 346.3	417.0 ± 326.0	0.104
Drainage volume on POD0 (ml)	128.5 ± 86.1	124.5 ± 106.6	0.605

BMI, Body mass index; CCI, Charlson comorbidity index; INR, International normalized ratio; POD0, postoperative day 0

*Represents for statistically different (*P* < 0.05)

**Table 4:** Postoperative outcomes by ambulation timing

	EA group	DA group	*P*
Variables	*N* = 326	*N* = 326	Value
Postoperative RBC count (×10^12^/L)	4.2 ± 0.5	4.3 ± 0.5	0.266
Postoperative HB (g/L)	128.9 ± 15.1	131.2 ± 15.0	0.079
First ambulation timing (hour)	23.5 ± 15.3	66.7 ± 13.2	< 0.001*
AEs	111 (34.0%)	150 (46.0%)	0.002*
Reoperation	1 (0.3%)	2 (0.6%)	1.000
Overall Complications	52 (16.0%)	94 (28.8%)	< 0.001*
*Medical complications*	45 (13.8%)	77 (23.6%)	0.002*
Urinary retention	6 (1.8%)	14 (4.3%)	
Deep vein thrombosis	4 (1.2%)	7 (2.1%)	
Nausea/vomiting	8 (2.5%)	12 (3.7%)	
Urinary Infection	2 (0.6%)	7 (2.5%)	
Acute cerebral infarction	4 (1.2%)	3 (0.9%)	
Pneumonia	3 (0.9%)	4 (1.2%)	
Delirium	3 (0.9%)	8 (2.5%)	
Acute myocardial infarction	1 (0.3%)	3 (0.9%)	
Constipation	16 (4.9%)	33 (10.1%)	
Arrhythmias	1 (0.3%)	4 (1.2%)	
*Surgery-related complication*	8 (2.5%)	10 (3.1%)	0.812
Total LOS	13 [11, 16]	14 [12, 17]	0.001*
Postoperative LOS (day)	7 [5, 9]	7 [6, 10]	0.073
Prolonged LOS	78 (23.9%)	96 (29.4%)	0.132
90-day Readmission	12 (3.7%)	19 (5.8%)	0.269

RBC, red blood cell; HB, hemoglobin; AEs, adverse events; LOS: length of hospital stay

*Represents for statistically different (*P* < 0.05)

### Risk factors for postoperative AEs

Seven risk factors (age, INR, operative time, drainage volume on POD0, number of fused segments, delayed ambulation, and diabetes) with a P value less than 0.1 were identified in the univariate analysis and then included in the multivariate analysis. Multivariate logistic regression identified older age (odds ratio [OR] 1.08 95% confidence interval [CI] 1.05–1.11, *p* < 0.001), operative time (OR 1.006 95%CI 1.003–1.009, *p* < 0.001), diabetes (OR 1.44 95%CI 1.02–2.04, *p* = 0.042) and delayed ambulation (OR 1.71 95%CI 1.22–2.39, *p* = 0.002) as independent risk factors for postoperative AEs (Table 5).

**Table 5:** Multivariate logistic regression for postoperative AEs.

Risk factors	OR (95% CI)	*P*-value
Age (yr)	1.08 (1.05–1.11)	<0.001*
INR	2.92 (0.54–15.90)	0.216
Operative time	1.006 (1.003–1.009)	<0.001*
Drainage volume on POD0 (ml)	1.001 (0.999–1.003)	0.212
fused segments	1.25 (0.82–1.93)	0.303
Delayed ambulation	1.71 (1.22–2.39)	0.002*
Diabetes	1.44 (1.02–2.04)	0.041*

AEs, Adverse events; INR, international normalized ratio; POD0, postoperative day 0

*Represents for statistically different (*P* < 0.05)

### Risk factors for delayed ambulation

In the univariate analysis, age, INR, number of fused segments, intraoperative EBL, operative time, drainage volume on POD0, CHD, current smoker and drinker were significantly different between the two groups with a p value below 0.05 and were select for multivariate analysis. Multivariable analysis identified older age (OR 1.06, *p* < 0.001) and intraoperative EBL (OR 1.001, *p* = 0.001), higher INR (OR 5.20, *p* = 0.032) as significant independent risk factors for DA (Table 6).

**Table 6:** Multivariate logistic regression for delayed ambulation

Risk factors	OR (95% CI)	*P*-value
Age(yr)	1.06 (1.04–1.09)	< 0.001*
INR	5.20(1.16–23.35)	0.032*
Number of fused segments	1.20 (0.98–1.46)	0.077
Intraoperative EBL (ml)	1.001 (1.000-1.002)	0.001*
Operative time (min)	1.001 (0.998–1.004)	0.396
Drainage volume on POD0	1.001 (0.999–1.002)	0.510
CHD Smoker Drinker	0.743(0.54–1.03) 1.55(0.83–2.88) 1.42(0.67–3.01)	0.074 0.171 0.365

INR, International normalized ratio; EBL, estimated blood loss; CHD, coronary heart disease

*Represents for statistically different (*P* < 0.05)

## Discussion

Early rehabilitation is an important component of the current multidisciplinary ERAS protocol for the management of patients undergoing lumbar fusion surgery[12, 18]. However, early ambulation was identified as an intervention with the lowest levels of adherence due to postoperative pain and concerns about displacement of implant and surgical site bleeding, especially in the elderly patients [19]. Our matched cohort study revealed that delayed ambulation was an independent risk factor of postoperative AEs. Furthermore, age, preoperative INR, and intraoperative EBL were associated with delayed ambulation.

Elderly patients are most likely to develop postoperative adverse events than younger patients. In previous study, age was identified as an independent risk factor for potential postoperative complications [20]. Early ambulation helps patients recover physiological function faster, reduce the time in bed and incidence of complications. In a retrospective study conducted by Huang et al., early ambulation within 24 h after total knee arthroplasty is shown to be associated with lower incidence of deep vein thrombosis and pulmonary infection[11]. These findings were also demonstrated in another study, in which the rate of complications was reduced by up to 23% for early ambulation patients underwent posterior spinal fusion surgery[21]. Furthermore, a randomized controlled trial demonstrated that the implement of early ambulation reduced pulmonary complication by 6% in colorectal surgery [22]. These results were further proven in our study. In the current study, patients in the DA group had higher rates of urinary retention and constipation. This may be because delayed walking can delay the recovery of many organ functions, such as digestive and bladder function, which can result in prolonged foley use and decreased bowel motility [23].

Early ambulation pathway was proven to reduce LOS by accelerating recovery in spine surgery [24], which was consistent with our results. Shorter hospital stay lighten the burden for the family, hospital and healthcare system. Early ambulation is a crucial component of ERAS pathways. In a study of 60 consecutive patients after elective major spinal surgery, ERAS pathway led to an average of 2.8 days reduction of LOS and reduced the cost by 29% compared with the traditional postoperative care[25]. Similarly, A retrospective cohort study on cesarean showed that the ERAS pathway resulted in the decrease of postoperative LOS by 7.8% (4.86 h) and the decrease of hospital costs by 8.4% ($642.85) per patient [26]. A systemic review concluded that the implement of early ambulation pathway reduced the LOS by 3.5 days and led to significant savings of hospital costs for patients going through pancreaticoduodenectomy [27]. Although the cost data was not evaluated in this study, it can be safely concluded from previous studies that reduced LOS can make a great contribution to healthcare cost savings.

With all the positive outcomes associated with early ambulation, it is vitally important to determine means to realize it. Nutritional support, normothermia maintenance and patient education were proven to improve the patient’s compliance with early ambulation after surgery [28]. Our study identified that younger age, lower INR and less EBL led to better compliance with early ambulation. Intraoperative antifibrinolytic agents, modified anesthesia techniques, and proper preoperative planning were associated with less blood loss in spine surgery, which can increase the patient’s compliance with early ambulation[29]. The aforementioned means could be used to prevent delayed ambulation in the future. Additionally, further investigation is needed to understand the relation between the use of anticoagulants and the risk of surgical complications in spinal surgery.

There were several limitations to the current study. First, this was a retrospective cohort study. However, due to ethical challenges and the lack of mobilization guidelines, it is difficult to conduct a prospective trial. Second, our study population was from a single center. Although our study population was larger than previous study, multicenter study with larger sample sizes may contribute to better evaluate the effect of delayed ambulation. Third, hospital costs data was not collected and analyzed in this study. Postoperative ambulation timing can be influenced by a patient’s baseline ambulatory status, which was not included in our analysis. Despite the aforementioned limitations, our study also had several strengths. To our knowledge, this is the first large-sample study identifying the benefit and safety of ambulation within 48 h after TLIF surgery in elderly patients. Additionally, our study matched the patients in the EA and DA groups by age, gender and number of fused segments, thus the confounders were less likely to infect the comparison of the two groups.

## Conclusion

Delayed ambulation was an independent risk factor for postoperative AEs after TLIF in elderly patients. Older age, increased intraoperative blood loss and worse coagulation function were associated with delayed ambulation. Future directions should focus on reducing the impact of these factors on postoperative ambulation timing.

## Data Availability

The underlying data supporting the results of this study could be obtained by contacting the corresponding author.

## Abbreviations

TLIF: Transforaminal Lumbar Interbody Fusion
Aes: Adverse Events
LOS: Length Of Hospital Stay
ERAS: Enhanced Recovery After Surgery
EA: Early Ambulation
DA: Delayed Ambulation
RBC: Red Blood Cells
INR: International Normalized Ratio
EBL: Estimated Blood Loss
POD0: Postoperative Day 0
CHD: Coronary Heart Disease
